# The effect of Orem's Self‐Care Deficit Theory–based care during pregnancy and postpartum period on health outcomes: A systematic review and meta‐analysis

**DOI:** 10.1111/ijn.13300

**Published:** 2024-08-29

**Authors:** Maide Nur Tümkaya, Kafiye Eroğlu, Zekiye Karaçam

**Affiliations:** ^1^ Faculty of Health Sciences Halic University Istanbul Turkey; ^2^ Graduate School of Health Sciences Koc University Istanbul Turkey; ^3^ Faculty of Health Sciences Atlas University Istanbul Turkey; ^4^ Faculty of Health Sciences Aydın Adnan Menderes University Aydın Turkey

**Keywords:** Orem, postpartum, pregnancy, Self‐Care Deficit Theory, women's health

## Abstract

**Aim:**

To examine the effect of the provision of care on health outcomes when provided based on Orem's Self‐Care Deficit Theory during perinatal period.

**Background:**

The perinatal period is a process with multidimensional care needs for the mother, baby and family. Care based on nursing theories can improve the quality of perinatal care.

**Design:**

A systematic review and meta‐analysis.

**Review methods:**

Studies on this topic from 2006 to 2020 have been accessed by nine database searches. The methodological quality of the studies was assessed using the Critical Appraisal Checklists for experimental and quasi‐experimental studies, developed by the Joanna Briggs Institute. This study was conducted by following the Preferred Reporting System for Systematic Reviews and Meta‐Analyses.

**Results:**

This systematic review included nine studies with a total sample size of 839 women. These studies showed that care based on Orem's theory significantly helped prolong the mean duration of pregnancy, reduced the incidence of preterm labor, improved hygiene behaviour and increased empowerment. Concerning the perinatal period, this intervention significantly improved self‐care and readiness for hospital discharge, adaptation and breastfeeding self‐efficacy and reduced nipple pain and pain due to abdominal distension.

**Conclusions:**

The implementation of this theory into care during the perinatal period can contribute to improved health outcomes.

**Summary statement:**

What is already known about this topic?

- The quality of pregnancy and postpartum care is important for the maternal health.
- The use of nursing theories—which nursing knowledge is produced—improves care quality and ensures that care is organized systematically.

What this paper adds?

- There is insufficient research that supports Orem's theory of self‐care deficit in pregnancy and postpartum care and also no systematic review about this topic, but the current meta‐analysis showed that postnatal and prenatal treatments based on Orem's theory have a positive impact on the self‐care agency.

The implications of this paper:

- The use of Orem's theory helps nurses understand women and their needs during pregnancy and postpartum periods.
- Integrating Orem's theory of self‐care deficit into pregnancy and postpartum nursing care services can help increase women's self‐care power.

## 1 INTRODUCTION

Maternal health refers to women's health during pregnancy, childbirth and the postpartum period. It is necessary to optimize the health and well‐being of women and their babies and help parents build favourable experiences during such periods. Maternal mortality rate significantly varies by Sustainable Development Goals region. Poor quality of care, delays in receiving care and inability to access safe, high‐quality and affordable sexual and reproductive health services are all causes of death (World Health Organization, 2023a). About 287 000 women died from preventable causes related to pregnancy and childbirth in 2020. Maternal mortality has decreased by 34% between 2000 and 2020, but when averaging reduction rates between 2016 and 2022, the figures have remained stable. United Nations Children's Fund (UNICEF), World Health Organization (WHO) and other partnering organizations are collaborating closely with country governments and other partners to accelerate progress in maternal and newborn health (UNICEF, 2023; World Health Organization, 2023b). New strategies are being developed through joint targets developed by the Every Newborn Action Plan and Ending Preventable Maternal Mortality groups to ensure that every pregnant woman receives essential interventions, such as four or more antenatal care visits, childbirth assisted by a skilled birth attendant and postnatal care for both mother and infant within 2 days of birth (World Health Organization, 2023b). Therefore, comprehensive and qualified care and training services have been implemented on a global scale; maternal health is guaranteed by laws and regulations, national health policies have been established (Coskun, 2016; Peahl et al., 2020) and pregnancy and postpartum follow‐up and care management guidelines (World Health Organization, 2017) have been put into service. In this context, it is also noted that the provision of high‐quality and safe care before, during and after birth shall improve and protect the perinatal health of the mother and the newborn (Bellerose et al., 2022; World Health Organization, 2019).

As it is known, psychological and physical changes occur in the prenatal and postnatal periods of women. During these processes, women may have difficulties in maintaining their daily life activities and performing self‐care. Therefore, the daily life needs of women should be met on time and with quality systematic nursing care during this process (Dipietro et al., 2019; Nazari et al., 2021). Thus, the woman can meet both her care and the needs of her baby without the need for help.

Psychological and physical problems before and after delivery may develop when the woman cannot fulfill her self‐care needs (Kilic & Erci, 2017; Lambermon et al., 2020). Inadequacies in maintaining self‐care during these periods unfavourably affect the physical and emotional health of women, the efficacy of infant care, maternity behaviour and social relationships (Aktas & Karacam, 2015; Jikijela et al., 2018; McCarter et al., 2022; Sis Celik & Aksoy Derya, 2019). Adequate self‐care agency, on the other hand, contributes positively to the improvement of physiological and psychological health, while helping the mother to assume an active role in infant care (Jikijela et al., 2018; Kaya Odabas & Taspinar, 2020; McCarter et al., 2022). Therefore, using the Orem Self‐Care Deficit Theory to address the lack of self‐care during these periods may be beneficial for maternal health.

According to Orem's Self‐Care Deficit Theory, the concept of self‐care is defined as activities aimed at promoting and protecting an individual's health (Mceven & Wills, 2014). In literature, studies show that providing care based on Orem's Self‐Care Deficit Theory is associated with improved maternal health (Keles & Eroğlu, 2023; Lambermon et al., 2020). However, a systematic review or meta‐analysis that synthesizes the findings of such studies to provide a higher level of evidence has not been found in the literature. As a result, we aimed to conduct this study to obtain the combined results of nursing interventions based on Orem's theory, which can help increase the quality of care in the pregnancy and postpartum period in terms of women's physical and mental health outcomes.

## 2 AIMS AND RESEARCH QUESTIONS

This systematic review aimed to determine the effect of care based on Orem's Self‐Care Deficit Theory on the physical and mental health outcomes in women, when provided during pregnancy and the postpartum period. The questions to be answered in the study were as follows:

- What is the effect of care provided during pregnancy based on Orem's Self‐Care Deficit Theory on the physical and mental health outcomes of pregnant women?
- What is the effect of care based on Orem's Self‐Care Deficit Theory on the physical and mental health outcomes of women when provided during the postpartum period?

## 3 METHODS

### 3.1 Design

This is a systematic review and meta‐analysis study. The principles of the Preferred Reporting Items for Systematic Review and Meta‐analysis (PRISMA) Statement were followed, and the relevant checklist was used for the development of the study protocol, the conduct of the study and the drafting of the manuscript (Page et al., 2021).

### 3.2 Protocol and registiration

To prevent duplications and compare with completed studies during the planning stage, the protocol of this study was registered on the PROSPERO database (date: 4 February 2022; registration no.: CRD42022300618). The review of the literature, the selection of articles, the data extraction and the quality assessment of the included articles were performed independently by two of the researchers (M.N.K and K.E.) to avoid the risk of any potential bias. Each step during this process was reviewed at meetings with the participation of all three researchers to achieve consensus. This research was conducted by two professors, one of whom is a PhD student in the field of obstetrics and gynecology, and has experience in systematic review and meta‐analysis.

### 3.3 Eligibility criteria

Studies eligible for this systematic review were selected according to the following criteria (PICOS):

Study population: Primipary or multiparous pregnant and postpartum women.
Intervention: The provision of care based on Orem's Self‐Care Deficit Theory.
Comparison: Standard care.
Outcomes: Physical and mental health outcomes were selected for evaluation. Concerning pregnancy, physical health outcomes included the duration of pregnancy as defined in studies, blood pressure, weight gain, proteinuria, hygiene behaviour, the self‐care agency score and mental health outcomes such as empowerment. Concerning the postpartum period, physical health outcomes included discharge readiness; self‐care agency scores; breastfeeding; the duration of exclusive breastfeeding; breastfeeding difficulties; blood pressure; body weight; pain in the shoulder, nipple and abdomen; and mental health outcomes such as adaptation, depression and self‐efficacy.
Study design: experimental and quasi‐experimental studies published in English and Turkish. Because the pilot study revealed that there were a few studies in the literature and that they were carried out in recent years, we did not specify a time frame for the years of study conduct in the eligibility criteria. Studies on this topic from 2006 to 2020 have been accessed.

We decided to exclude studies with no clear methodology or no available full text, studies with observational design, animal experiment studies and studies that employed Orem's Self‐Care Deficit Theory but not during pregnancy and the postpartum period.

### 3.4 Search strategy

The literature search for this study was performed in the period between 30 December 2021 and 1 January 2022, in electronic databases including PubMed, EBSCO (Medline, CINAHL), Embase (OVID) Web of Science, PsycINFO (all via Ovid SP), Scopus, Cochrane, Türkiye Klinikleri (Turkish reference directory), TR Dizin (a directory) and Council of Higher Education—National Thesis Center. Then, the research was completed in March 2022. The keywords used in the review of international databases included (pregnancy OR pregnant OR postpartum OR puerperium OR postnatal) AND (“Orem's Self‐Care deficit theory” OR “Orem Self‐Care Model” OR “Orem Self Care Model” OR “Orem's self‐care”). The search strategy for the review is presented in Supplementary file 3. A grey literature search in Google Scholar and National Thesis Center databases and a manual search of study references were also conducted.

### 3.5 Study selection

**FIGURE 1:**
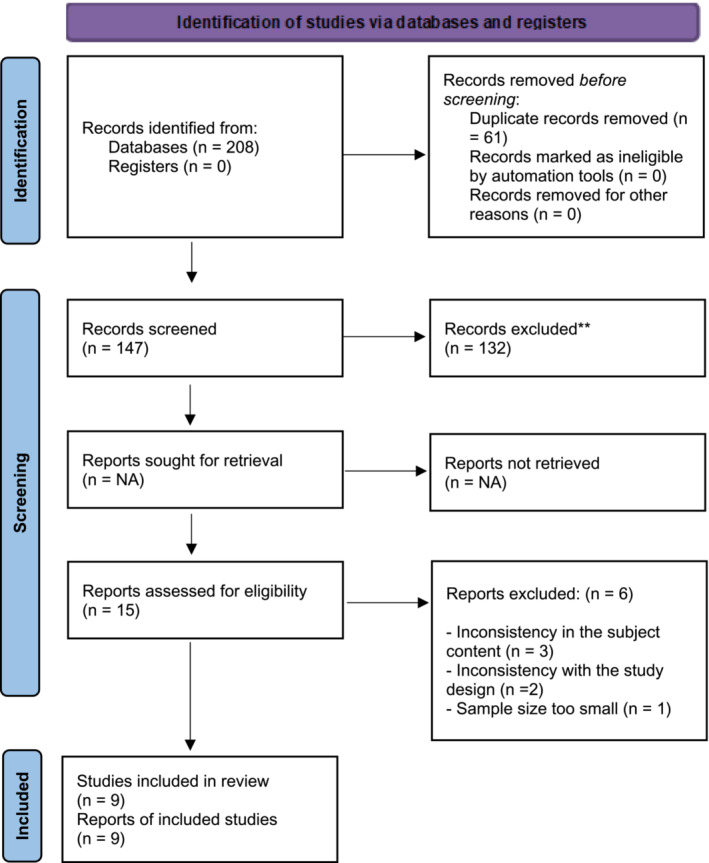
PRISMA flow diagram.

Experimental and quasi‐experimental studies using Orem's Self‐Care Deficit Theory in pregnancy and postpartum care services were selected for this systematic review and meta‐analysis. EndNote version 20.1, a reference management program, was used to review all citations and remove duplicates from databases. Three researchers used Covidence to undertake independent study selection and data extraction, remaining blind to each other's decisions but not to journal titles, study authors, or institutions. To assess eligibility, the researchers screened the publications based on the inclusion and exclusion criteria, resolving any inconsistencies through group discussions. The PRISMA flowchart (Figure 1) was used to document and report the screening results.

### 3.6 Data extraction

To ensure the validity and high quality of the data, standardized and predefined data extraction forms were utilized (Table 1). Collected information via this data extraction tool included author information; the year of publication; study site; study design; sample size; study group characteristics (as described in studies); the year of obtaining data in studies; the time, duration and mode of applied intervention; comparison group characteristics (as described in studies); and physical and emotional problems (as defined in studies). The data extraction process was carried out by the first and the second authors independently.

**TABLE 1:** The list of studies included in the systematic review.

Author(s), year, country	Study design, year	Data collection tool	Sample size, groups and participant characteristics, maternal age	Characteristics of the intervention	Main outcomes (intervention and control)
Caliskan and Sahin (2020) Turkey	RCT, 2019	GHBI	Intervention group: 33 Control group: 34 Pregnant women, who presented to the pregnancy outpatient clinic Mean age (year): 28.12 ± 4.85 and 28.26 ± 4.80	A 25–30 min training program was applied once; a training brochure was provided, and a 10‐min phone call was made 2 weeks after the training.	GHBI scores: 69.54 ± 7.75 and 62.76 ± 7.16
Capik et al. (2015) Turkey	Quasi‐experimental, 2011	Questionnaire, postpartum self‐evaluation questionnaire*	Intervention group: 55 Control group: 56 Primipara women who had a vaginal delivery at term Age: > 18	Nursing care, 6 home visits once a week starting on the 3rd postnatal day	Postpartum adaptation scores: 252.91 ± 10.69 and 308.39 ± 7.64. Number of nursing diagnoses: 28 and 345
Houshmandpour et al. (2019) Iran	RCT, 2017	Self‐care questionnaire Pregnant Women's Empowerment Questionnaire for pregnant women and Self‐care Agency Scale (SCAS)	Intervention group: 37 Control group: 37 Primiparous pregnant women ‐‐	A training program of one 30‐min session a week for 6 weeks was conducted. Assessments were performed before, immediately after and 3 weeks after the training.	SCAS scores: 320.29 ± 20.18 and 263.37 ± 21.19 (3rd week posttest) Empowerment scores: 3rd week posttest: 98.83 ± 4.31 and 56.10 ± 9.79
Kilic and Erci (2017) Turkey	Quasi‐experimental, 2010–2011	High‐Risk Pregnancy Follow‐up Form, SCAS	Intervention group: 40 Control group: 40 Pregnant women diagnosed with preterm birth risk Age (year): 27.05 ± 5.27 and 27.62 ± 5.02	During the hospital stay, bedside visits for pregnant women three times a week and intervention according to the diagnosis. After the first three visits, pregnant women were visited two or three times a week depending on the condition. The purpose was to identify and meet the care needs of women.	SCAS scores: 121.17 ± 13.58 and 96.62 ± 18.60 Universal self‐care diagnoses: 45 and 105 Self‐care diagnoses in health deviations: 58 and 134 Developmental self‐care diagnoses: 53 and 155. Number of problems experienced: 66 and 99
Kilic and Inanc (2006) Turkey	Quasi‐experimental, 2004–2005	SCAS, Form for determining the health problems experienced	Intervention: 40 Control: 40 Primiparous women who gave birth by cesarean section, Age: > 19 years	A total of 3 training sessions, each lasting 30–40 min, in the 30th and 35th weeks of pregnancy and on the day before the cesarean section	SCAS mean scores: 91.6 ± 12.8 and 83.2 ± 8.5 Incision pain: 35 and 37 Back‐shoulder pain: 9 and 11 Breastfeeding problems: 7 and 13 Pain due to distention: 4 and 12 Nipple pain: 11 and 26
Nazik and Eryilmaz (2013) Turkey	Quasi‐experimenta, 2008	Postpartum follow‐up form SCAS	63 primiparous who had vaginal delivery, Age range: 19–36 years	Nursing care started before the hospital discharge after birth and continued until the end of the postpartum period (duration: 7 weeks).	SCAS: Pretest: 97.13 ± 17.20 Posttest: 114.44 ± 13.72
Rezaeean et al. (2020) Iran	RCT, 2015	Holbrook's Preterm Delivery Screening Questionnaire Hart Prenatal Care Actions Scale	Intervention: 88 Control: 88 Women in the 24th–26th weeks of pregnancy, at risk of premature birth, Age: 28.61 ± 6.21 and 28.38 ± 6.62	Self‐care training: 45–60‐min face‐to‐face individual training for three consecutive weeks, followed by a follow‐up call on the phone by the 40th week latest.	The frequency of preterm birth: 6 (6.80%) and 18 (20.50%). Mean duration of pregnancy: 38.97 ± 1.45 and 38.27 ± 2.61
Shobeiri et al. (2016) Iran	RCT, 2014	A demographic information‐care ability assessment questionnaire was prepared by the researcher based on Orem's theory.	Intervention: 30 Control: 30 Pregnant women diagnosed with preeclampsia Intervention:28.9 ± 7.3 Control: 27.3 ± 6.6 Gestational age (days): 201.4 ± 28.9 and 246.9 ± 21.9	Training sessions of 20–30 min, 2 times a week for 4 weeks during pregnancy	**During pregnancy:** Blood pressure: 13.53 ± 1.3 and 14.2 ± 1.4 Weight: 76.2 ± 11.8 and 76.2 ± 19.4 Proteinuria: 1.5 ± 0.8 and 1.2 ± 0.4 **Postpartum period:** Blood pressure: 11.7 ± 8.3 and 12.4 ± 0.8 Weight: 66.6 ± 12.9 and 62.4 ± 11.6 Proteinuria: 1.2 ± 0.5 and 1.5 ± 0.8 About self‐care knowledge scores: 9.5 ± 1.8 and 3.5 ± 2.3 Attitude scores: 6.1 ± 0.4 and 11.4 ± 4.6 Skill scores: 10.8 ± 0.4. and 5.7 ± 2.2 Body image perception scores: 8.6 ± 1.7 and 11.3 ± 3.1 Self‐confidence scores: 10.4 ± 1.8 and 14.7 ± 4.1
Sahin and Yavan (2015) Turkey	RCT, 2014	BSES, PRDBS, SCAS, EPDS	Intervention: 64 Control: 64 Women who have given vaginal and cesarean delivery Age: Intervention: 30.82 ± 4.90 (20–41) Control: 31.71 ± 6.02 (17–42)	Individual training before hospital discharge for postpartum women (women's self‐care and newborn care) and then, if needed, 6‐month telephone counseling service	**Duration of breastfeeding:** 4.50 ± 1.77 and 3.97 ± 2.07 BSES scores: 6th week: 61.17 ± 6.18 and 51.78 ± 7.31 SCAS scores: 6th week: 102.19 ± 25.85 and 85.59 ± 26.72 PRDBS scores: 176.16 ± 12.57 and 150.34 ± 25.78 EPDS scores: 5.00, min‐max: 3.00–7.00 and 6.00, min–max: 4.00–8.00

Abbreviations: RCT, randomized controlled trial; GHBI, Genital Hygiene Behaviors Inventory; SCAS, Self‐care Agency Scale; BSES, Breastfeeding Self‐Efficacy Scale. PRDBS, Perceived Readiness for Discharge after Birth Scale; EPDS, Edinburgh Postpartum Depression Scale.

### 3.7 Methodological quality assessment of studies

The articles included in this systematic review were examined for their methodological quality, using the JBI Critical Appraisal Checklist for Randomized Clinical Trials and Quasi‐Experimental Studies published by the Joanna Briggs Institute (Table 2). The checklist for randomized controlled trials consisted of 13 items, and the checklist for quasi‐experimental studies consisted of nine items. These checklists include questions regarding blinding, randomization, appropriate study design, measurement reliability, similarity of groups, allocation, intervention and completion of follow‐ups (Tufanaru et al., 2020). One of the following responses including “yes,” “no,” “unclear” or “not applicable” were attained for each item. The methodological quality level of the studies included in this research was evaluated as “mediocre,” “moderate” or “good” if the answer was “yes” to < 50% of the items, “yes” to 51–80% of the items and “yes” to more than 80% of the checklist items, respectively.

**TABLE 2:** Quality assessment scores of studies.

Studies	JBI critical appraisal checklist questions for randomized controlled studies													Quality score of the study
	Q1	Q2	Q3	Q4	Q5	Q6	Q7	Q8	Q9	Q10	Q11	Q12	Q13	
Caliskan and Sahin (2020)	Y	N	Y	N	N	N	Y	Y	Y	Y	Y	Y	Y	Moderate (69.2%)
Houshmandpour et al. (2019)	U	N	Y	N	N	N	Y	Y	Y	Y	Y	Y	Y	Moderate (61.5%)
Rezaeean et al. (2020)	N	N	Y	Y	N	N	Y	Y	Y	Y	Y	Y	Y	Moderate (69.2%)
Shobeiri et al. (2016)	N	N	Y	N	N	N	Y	Y	Y	Y	Y	Y	Y	Moderate (61.5%)
Sahin and Yavan (2015)	U	N	Y	N	N	N	Y	Y	Y	Y	Y	N	Y	Moderate (53.8%)
**Question quality score**	20.0%	0.0%	100%	20.0%	0.0%	0.0%	100%	100%	100%	100%	100%	80.0%	100%	
**JBI critical appraisal checklist questions for quasi‐experimental studies**														
Capik et al. (2015)	Y	Y	Y	Y	Y	Y	Y	Y	Y					Good (100%)
Kilic and Erci (2017)	Y	Y	Y	Y	Y	Y	Y	Y	Y					Good (100%)
Kilic and Inanc (2006)	Y	Y	Y	Y	Y	Y	Y	Y	Y					Good (100%)
Nazik and Eryilmaz (2013)	Y	N/A	N/A	N	Y	Y	Y	Y	Y					Moderate (66.7%)
**Question quality score**	100%	75.0%	75.0%	75.0%	100%	100%	100%	100%	100%					

Abbreviations: Q, question; Y, yes; N, no; N/A, not applicable; U, unclear.

The quality assessment process was carried out independently by the first and second authors. Then, all three researchers discussed, resolved disagreements and created a one‐sheet report of findings at a meeting.

### 3.8 Data synthesis

In this systematic review, meta‐analysis and narrative synthesis methods were used. Five of these studies had data that could be included in the meta‐analysis (on self‐care agency during pregnancy and the postpartum period). Seven studies had other outcomes data reported by narrative synthesis. The effect size was calculated by meta‐analysis for each outcome variable reported in one or more studies. The meta‐analysis was performed using RevMan 5.4.1 (The Nordic Cochrane Center, Copenhagen, Denmark). A 95% confidence interval (CI) and the odds ratio (OR) were calculated for categorical variables. The mean difference (MD) and standardized mean difference (SMD) were calculated for continuous variables. SMD was calculated for the measurement outcomes made with different measurement tools, and MD was calculated for the measurement outcomes made with the same measurement tools. In addition, subgroup analyses were performed to determine the effects of the pregnancy and postpartum periods on self‐care agency. Heterogeneity between studies was assessed using the *χ* ^2^ test and *I* ^2^ statistic. *I* ^2^ is 0%–40% indicated might not be important, *I* ^2^ is 30%–60% represented moderate heterogeneity, *I* ^2^ is 50%–90% represented substantial heterogeneity and *I* ^2^ is 75%–100% indicated considerable heterogeneity (Deeks et al., 2021). In this study, a random‐effects model was used to incorporate heterogeneity among studies included in the meta‐analysis. All values were calculated from two‐tailed tests, and a *p*‐value of < 0.05 was considered statistically significant.

## 4 RESULTS

### 4.1 Search results

The literature search revealed a total of 208 records for published articles. When duplicates were excluded and eligible articles were selected based on the review of headings and abstracts, 14 articles remained. After the examination of the full texts of these studies according to the inclusion criteria, nine studies were included in this systematic review (Figure 1).

### 4.2 Characteristics of studies and participants

Five of the studies included in this research were randomized controlled trials (Caliskan & Sahin, 2020; Houshmandpour et al., 2019; Rezaeean et al., 2020; Sahin & Yavan, 2015; Shobeiri et al., 2016), and four were quasi‐experimental studies (Capik et al., 2015; Kilic & Erci, 2017; Kilic & Inanc, 2006; Nazik & Eryilmaz, 2013). The studies were conducted in the years between 2004 and 2019. Of the studies, three were conducted in Iran (Houshmandpour et al., 2019; Rezaeean et al., 2020; Shobeiri et al., 2016) and six were conducted in Turkey (Caliskan & Sahin, 2020; Capik et al., 2015; Kilic & Erci, 2017; Kilic & Inanc, 2006; Nazik & Eryilmaz, 2013; Sahin & Yavan, 2015). The total sample size of the studies was 839 (intervention group: 387 participants; control group: 389 participants; pre‐test and post‐test: 63 participants). Five of the studies were conducted with pregnant women and four with postpartum women. In one of the studies, pregnant women with preeclampsia with preterm birth risk were included. Two of the studies included pregnant women diagnosed with preterm birth risk. The participant age in the included studies was 17 years and above (Table 1).

### 4.3 Characteristics of intervention

Orem's theory was used in six studies (Caliskan & Sahin, 2020; Houshmandpour et al., 2019; Kilic & Inanc, 2006; Rezaeean et al., 2020; Sahin & Yavan, 2015; Shobeiri et al., 2016) for implemeting training programs for women and in three studies (Capik et al., 2015; Kilic & Erci, 2017; Nazik & Eryilmaz, 2013) for nursing care and follow‐ups. More comprehensive information about the interventions made in the studies is given in Table 1. Interventions were administered in a hospital setting in five studies, at home in one study and both at home and in the hospital setting in three studies. In three studies with postpartum women, interventions were continued for 6 weeks (Capik et al., 2015; Houshmandpour et al., 2019; Sahin & Yavan, 2015). The duration of training sessions was reported in the other studies as varying from 25 to 60 min (Caliskan & Sahin, 2020; Kilic & Erci, 2017; Kilic & Inanc, 2006; Nazik & Eryilmaz, 2013; Rezaeean et al., 2020; Shobeiri et al., 2016) (Table 1). Routine clinical practices were applied to the control groups.

### 4.4 Quality assessment results

The quality assessment scores of five randomized controlled experimental studies were moderate. Of the quasi‐experimental studies, three were good quality and one was moderate quality (Table 2).

### 4.5 Results on the effect of care based on Orem's Self‐Care Deficit Theory on the health outcomes in pregnancy

The effect of care based on Orem's Self‐Care Deficit Theory on preterm birth was reported by one study (Rezaeean et al., 2020). It was found that the probability of preterm labor was lower (OR: 0.28 [95% CI: 0.11–0.76], *Z* = 2.52, *p* = .01) in the intervention group. In addition, Rezaeean et al., 2020 reported that the mean gestational period of the experimental group was statistically significantly longer than the control group (MD: 0.70 weeks [95% CI: 0.08–1.32], *Z* = 2.20, *p* = .030).

Another study examined the effects of this intervention on blood pressure, body weight and proteinuria during pregnancy (Shobeiri et al., 2016). However, analyses revealed no statistically significant differences between the groups (MD: −0.70 [95% CI: −3.68 to 2.28], *Z* = 0.46, *p* = .650; MD: 0.00 [95% CI: −8.13 to 8.13], *Z* = 0.00, *p* = 1.00; MD: 0.30 [95% CI: −0.02 to 0.62], *Z* = 1.84, *p* = 0.07, respectively).

The effect of the intervention on genital hygiene during pregnancy was examined in one study included in this systematic review (Caliskan & Sahin, 2020). It was found that the experimental group experienced a statistically significant more favourable effect on genital hygiene behaviour compared to the control group (MD: 6.78 [95% CI: 3.20–10.36], *Z* = 3.72, *p* < .001). The effect of the intervention on pregnant women's empowerment scores was evaluated in a study (Houshmandpour et al., 2019), and a statistically significant result was found in favour of the experimental group (MD: 42.73 [95% CI: 39.16–46.30], *Z* = 23.45 *p* < .001).

A study with pregnant women at risk of preterm birth reported that, compared to the control group, there were fewer nursing diagnoses concerning the universal, developmental and health deviation self‐care domains in the intervention group and individuals in the intervention group experienced fewer health problems (Kilic & Erci, 2017).

### 4.6 Results on the effect of care based on Orem's Self‐Care Deficit Theory on self‐care agency during pregnancy and the postpartum period

**FIGURE 2:**
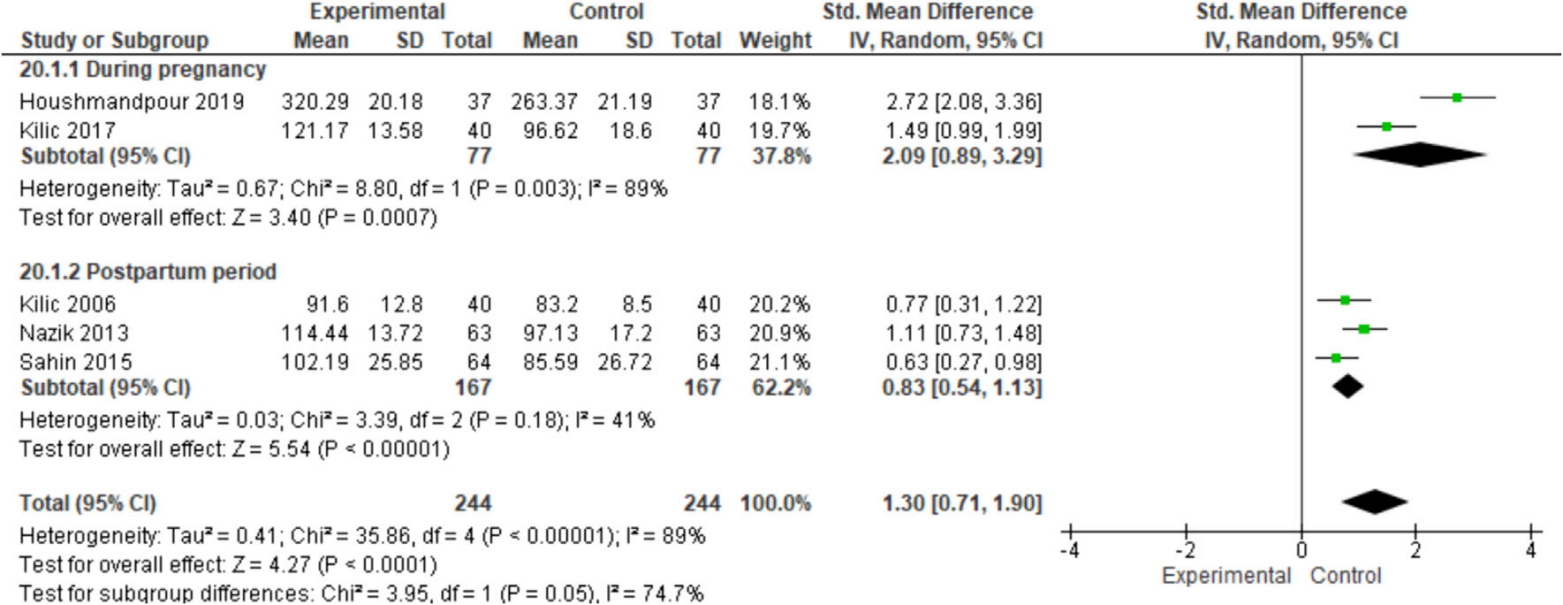
Forest plots on self‐care agency during pregnancy and the postpartum period. Abbreviations: Total, total sample size in group; IV, inverse variance; SD, standard deviation; 95% CI, 95% confidence interval.

Five studies included in the meta‐analysis examined the effect of interventions based on Orem's Self‐Care Deficit Theory on self‐care during pregnancy and the postpartum period (Houshmandpour et al., 2019; Kilic & Erci, 2017; Kilic & Inanc, 2006; Nazik & Eryilmaz, 2013; Sahin & Yavan, 2015). The meta‐analysis revealed that this intervention statistically significantly improved self‐care agency in the pregnancy and postpartum periods (SMD: 1.30, *Z* = 0.71 [95% CI: 0.71–1.90], *p* < .010, *I* ^2^ = 89%). This significant effect continued in the subgroup analysis for pregnancy and postpartum periods (SMD: 2.09, [95% CI: 0.89–3.29], *Z* = 3.40, *p* < .001, *I* ^2^ = 89%; SMD: 0.83, [95% CI: 0.54–1.13], *Z* = 5.54, *p* < .001, *I* ^2^ = 41%, respectively) (Figure 2). The heterogeneity between studies included in these analyses was substantial (*χ* ^2^ = 35.86, df = 4, *p* < .001, *I* ^2^ = 89%).

### 4.7 Results on the effect of care based on Orem's Self‐Care Deficit Theory on the health outcomes in postpartum periods

One study examined the effect of theory‐based intervention on hospital discharge readiness (Sahin & Yavan, 2015). The level of readiness for hospital discharge was statistically significantly higher in the experimental group compared to the control group (MD: 25.82 [95% CI: 18.79–32.85], *Z* = 7.20, *p* < .001). Another study that examined the effect of the intervention on postpartum adaptation (Capik et al., 2015) revealed that the intervention resulted in a favourable effect on the adaptation level of the experimental group (MD: −55.48 [95% CI: −58.94 to 52.02], *Z* = 31.41, *p* < .001).

The effects of interventions based on Orem's Self‐Care Deficit Theory on breastfeeding were examined in two studies (Kilic & Inanc, 2006; Sahin & Yavan, 2015;). Sahin and Yavan (2015) revealed that the breastfeeding self‐efficacy score of the experimental group was statistically significantly higher than that of the control group in one study (MD: 9.39 [95% CI: 7.04–11.74], *Z* = 7.85, *p* < .001), and the intervention was not effective on the duration of exclusive breastfeeding (MD: 0.53 [95% CI: −0.14‐1.20] *Z* = 1.56, *p* = .120). Kilic and Inanc (2006) showed that Orem's theory‐based care given to postpartum women statistically significantly reduced nipple pain (OR: 0.20 [95% CI: 0.08–0.53], *Z* = 3.27, *p* = .001).

The effect of the intervention based on Orem's Self‐Care Deficit Theory was examined on pain at the incision site, pain due to distension and shoulder and back pain after cesarean delivery in a study (Kilic & Inanc, 2006). The analysis showed that pain at the incision site and on the shoulder and the back was not statistically significantly different between groups (OR: 0.57 [95% CI: 0.13–2.55], *Z* = 0.74, *p* = .460; OR: 0.77, *Z* = 0.52, *p* = .061, respectively) However, it was found that the intervention reduced pain due to abdominal distension (OR: 0.26 [95% CI: 0.08–0.89], *Z* = 2.14, *p* = .030).

The effects of the intervention based on Orem's Self‐Care Deficit Theory on postpartum blood pressure, body weight and proteinuria were examined in a study included in this systematic review (Shobeiri et al., 2016). The analysis revealed a nonsignificant difference in those three variables between the experimental and control groups (MD: −0.70 [95% CI: −3.68 to 2.28], *Z* = 0.46, *p* = .650; MD: 4.20 [95% CI: −2.01 to 10.41], *Z* = 1.33, *p* = .180; MD: −0.30 [95% CI: −0.64 to 0.04], *Z* = 1.74, *p* = .080, respectively).

A study included in this systematic review reported that the postpartum depression scale scores were statistically significantly lower in the intervention group compared to the control group (Sahin & Yavan, 2015).

One of the studies included in this systematic review was conducted with women, who had a vaginal delivery. It was observed in that study that the group of women, who received care based on Orem's Self‐Care Deficit Theory, had fewer nursing diagnoses compared to the control group (Capik et al., 2015).

## 5 DISCUSSION

This systematic review conducted to determine the effects of interventions based on Orem's Self‐Care Deficit Theory on health outcomes of pregnant and postpartum women presents the results from pooled data of nine studies, which previously reported the effects of prepartum and postpartum interventions on maternal health. Our results are valuable because they provide important evidence‐based information that can be used to promote the health of pregnant and postpartum women.

Studies in the literature report that the self‐care needs of the mother are associated with emotional well‐being, as well as physical health, during pregnancy and in the first days after birth (Lambermon et al., 2020; Solhi et al., 2019). In this study, we found that nursing interventions based on Orem's Self‐Care Deficit Theory improved the self‐care capacity and empowerment of pregnant and postpartum women. This result is important for the promotion of maternal health.

This study showed that theory‐based interventions during pregnancy statistically significantly help prolong the mean duration of pregnancy and decrease the incidence of preterm labor. However, the results show that such interventions did not affect weight gain, proteinuria, or blood pressure. Although there are no similar studies in the literature to support our results, there are studies available showing the effect of care based on different theories/models on maternal health outcomes. One study reported that training, which was provided based on Roy's Adaptation Model, was associated with reductions in blood pressure in patients with gestational hypertension although it did not affect the duration of pregnancy (Amanak et al., 2019). However, the studies examining the effects of the provision of nursing theory–based care on pregnancy outcomes are very rare and there is a need for future studies for high levels of evidence.

This study has shown that care based on Orem's Self‐Care Deficit Theory favourably improves genital hygiene behaviour during pregnancy. Similarly, a study in the literature reported favourable effects of Pender's Health Promotion Model on women's attitudes, resulting in the stopping of vaginal douching (Mete et al., 2012). These results suggest that the use of nursing theory and models can be used to promote women's genital hygiene behaviour of women.

In this study, it has been found that care based on Orem's Self‐Care Deficit Theory favourably affects postpartum self‐care agency, adaptation and readiness for hospital discharge. However, another study reported that education based on Roy's Adaptation Model did not affect postpartum adaptation (Sercekus & Mete, 2010). These results may indicate differences between theories resulting in differences in their effects on postpartum adjustment. Therefore, future research is needed to obtain information on the subject matter.

Our study results revealed that the interventions based on Orem's Self‐Care Deficit Theory improved breastfeeding self‐efficacy scores and decreased nipple pain, but such interventions did not affect the occurrence of breastfeeding problems and the duration of exclusive breastfeeding. These indicate the favourable effects of theory‐based interventions on breastfeeding self‐efficacy and nipple pain. Similar to our findings, one study using Pender's Health Promotion Model (Sari & Altay, 2020) and another using Roy's Adaptation Model (Apay & Pasinlioglu, 2014) reported that such interventions favourably affected breastfeeding self‐efficacy and reduced breastfeeding problems. These results provide valuable contributions to the literature because they show the favourable effect of nursing theory‐based care on mothers' breastfeeding behaviour.

This study revealed that care based on Orem's Self‐Care Deficit Theory reduced pain due to abdominal distension after cesarean section. However, it did not affect blood pressure, changes in body weight, proteinuria, pain at the incision site and shoulder and back pain in the postpartum period. Similarly, a study reported that postpartum abdominal pain was significantly reduced when care was provided based on Roy's Adaptation Model to women, who gave birth by cesarean section (Apay & Pasinlioglu, 2014). These results provide important information showing that planned care based on a well‐established theory and a model can help alleviate potential postpartum problems. However, there is a need for further studies to obtain high‐level evidence on this subject matter.

Our study showed that nursing care based on Orem's Self‐Care Deficit Theory decreases the number of nursing diagnoses and reduces postpartum depression scale scores. Similar to our results, it was reported that the provision of postpartum care based on Roy's Adaptation model resolved or prevented the majority of postpartum problems (Apay & Pasinlioglu, 2014). In that study, in the experimental group, a total of 36 nursing diagnoses were made and 28 of them were resolved by the provided care. The majority of those diagnoses were resolved, including the risk of developing or transmitting infection, inadequate breastfeeding, interruption of breastfeeding, the sense of loneliness, lack of information (diet, hygiene, breastfeeding and family planning), social isolation and parental role conflict. When the number of nursing diagnoses in the last week of the postpartum period was compared between the experimental and control groups, the differences were statistically significant. Another study, where a postpartum intervention based on Roy's model was provided, reported that the depression scale scores of the experimental group were significantly lower compared to the control group in the first, second and third months after the intervention (Wang & Li, 2021).

### 5.1 Limitations

The study has some limitations. Firstly, most of the studies were conducted in Turkey and some of the meta‐analysis results were obtained from only a few studies and studies with small sample sizes. Because of the small number of research, it was not able to investigate subgroup analysis or metamorphosis to determine a likely source of heterogeneity to lower the *I* ^2^ index value. Secondly, heterogeneity between studies was high, which might have compromised the strength of the results. Therefore, to avoid the effect of heterogeneity between studies, the random effect model was chosen in the relevant meta‐analyses.

## 6 CONCLUSIONS AND IMPLICATION FOR PRACTICE, THEORY AND RESEARCH

This study revealed that nursing care based on Orem's Self‐Care Deficit Theory during pregnancy and postpartum periods is effective in reducing women's physical and mental health problems and improving self‐care. Based on these outcomes, the following might be suggested. (1) Nurses should benefit from Orem's Self‐Care Deficit Theory, as part of effective and quality nursing care practice, to achieve favourable health outcomes during pregnancy and the postpartum period. (2) Orem's Self‐Care Deficit Theory should be included in formal and non‐formal nursing education about pregnancy and postpartum care services. (3) Health institution managers should integrate the use of Orem's Self‐Care Deficit Theory into institutional care services and promote its use in pregnancy and postpartum care services. (4) Conduct more qualitative and quantitative studies that might provide comprehensive information to the literature and clinic about this theory that can improve the quality of care during pregnancy and the postpartum period, which are considered sensitive periods for mothers and babies.

### AUTHORSHIP STATEMENT

Study design: MNT, KE, ZK. Data collection: MNT, KE, ZK. Data analysis: MNT, KE, ZK. Study supervision: MNT, KE, ZK. Manuscript writing: MNT, KE, ZK. Critical revisions for important intellectual content: MNT, KE, ZK. All authors read and approved the final manuscript.

## CONFLICT OF INTEREST

The authors declare no conflict of interest.

## PROSPERO REGISTIRATION NUMBER

CRD42022300618.

## Supporting information


**Data S1.** Supporting Information

## Data Availability

The data that support the findings of this study are available from the corresponding author upon reasonable request.

## ENDNOTE

* For this scale score increases, the level of adaptation decreases.
