# Supplementary material for: The effect of Orem's Self‐Care Deficit Theory–based care during pregnancy and postpartum period on health outcomes: A systematic review and meta‐analysis
Source: Int J Nurs Pract. 2024 Aug 29;30(6):e13300. doi: 10.1111/ijn.13300 (PMC11608932; doi:10.1111/ijn.13300)
Supplement: Supplementary file 1 — Data S1. Supporting Information [file IJN-30-e13300-s001.docx]

**Pubmed**

("Orem's Self-Care deficit theory"[All Fields] OR "orem self care model"[All Fields] OR "orem self care model"[All Fields] OR "Orem's self-care"[All Fields]) AND ("Pregnancy"[MeSH Terms] OR ("Pregnancy"[MeSH Terms] OR "Pregnancy"[All Fields] OR "pregnancies"[All Fields] OR "pregnancy s"[All Fields]) OR ("gravidity"[MeSH Terms] OR "gravidity"[All Fields] OR "pregnant"[All Fields] OR "pregnants"[All Fields]) OR ("postpartum period"[MeSH Terms] OR ("postpartum"[All Fields] AND "period"[All Fields]) OR "postpartum period"[All Fields] OR "postpartum"[All Fields]) OR ("postpartum period"[MeSH Terms] OR ("postpartum"[All Fields] AND "period"[All Fields]) OR "postpartum period"[All Fields] OR "puerperium"[All Fields]) OR ("postnatal"[All Fields] OR "postnatally"[All Fields]))

12

**Cochrane Library**

#1

"Orem’s Self-Care deficit theory" OR "Orem Self-Care Model" OR "Orem Self Care Model" OR "Orem's self-care"

#2

pregnancy OR pregnant OR postpartum OR puerperium OR postnatal

#1 AND #2

12

**Scopus**

TITLE-ABS-KEY ( "Orem's Self-Care deficit theory" OR "Orem Self-Care Model" OR "Orem Self Care Model" OR "Orem's self-care" ) AND TITLE-ABS-KEY ( pregnancy OR pregnant OR postpartum OR puerperium OR postnatal )

17

**Web of Science**

#1

TS=("Orem’s Self-Care deficit theory" OR "Orem Self-Care Model" OR "Orem Self Care Model" OR "Orem's self-care")

#2

TS=(pregnancy OR pregnant OR postpartum OR puerperium OR postnatal)

#3

#1 AND #2

7

**CINAHL**

("Orem’s Self-Care deficit theory" OR "Orem Self-Care Model" OR "Orem Self Care Model" OR "Orem's self-care" ) AND ( pregnancy OR pregnant OR postpartum OR puerperium OR postnatal)

118

**PsycInfo**

( "Orem’s Self-Care deficit theory" OR "Orem Self-Care Model" OR "Orem Self Care Model" OR "Orem's self-care" ) AND ( pregnancy OR pregnant OR postpartum OR puerperium OR postnatal )

3

**Ovid MEDLINE(R) <1946 to December Week 3 2021>**

1 Pregnancy/ or pregnancy.mp. 979948

2 pregnant.mp. or Pregnant Women/ 181939

3 postpartum.mp. or Postpartum Period/ 70562

4 puerperium.mp. 8244

5 postnatal.mp. 107732

6 1 or 2 or 3 or 4 or 5 1083018

7 Orem Self Care deficit theory.mp. 1

8 Orem Self-Care Model.mp. 7

9 Orem Self Care Model.mp. 7

10 Orem's self-care.mp. 210

11 Orem self-care.mp. 15

12 7 or 8 or 9 or 10 or 11 222

13 6 and 12 13

**OVID**

1 ("Orem’s Self-Care deficit theory" or "Orem Self-Care Model" or "Orem Self Care Model" or "Orem's self-care").mp. [mp=title, abstract, full text, caption text] 138

2 (pregnancy or pregnant or postpartum or puerperium or postnatal).mp. [mp=title, abstract, full text, caption text] 140298

3 1 and 2 19

**Total Records: 206**

**After duplicate records are removed: 152**
